# Using re-randomisation designs to increase the efficiency and applicability of retention studies within trials: a case study

**DOI:** 10.1186/s13063-023-07323-y

**Published:** 2023-04-29

**Authors:** Beatriz Goulao, Anne Duncan, Karen Innes, Craig R. Ramsay, Brennan C. Kahan

**Affiliations:** 1 Health Services Research Unit, University of Aberdeen, Aberdeen, Scotland; 2 MRC Clinical Trials Unit at UCL, London, UK

**Keywords:** Trials, Trial methodology, Re-randomisation, Retention, SWAT (study within a trial)

## Abstract

**Background:**

Poor retention in randomised trials can lead to serious consequences to their validity. Studies within trials (SWATs) are used to identify the most effective interventions to increase retention. Many interventions could be applied at any follow-up time point, but SWATs commonly assess interventions at a single time point, which can reduce efficiency.

**Methods:**

The re-randomisation design allows participants to be re-enrolled and re-randomised whenever a new retention opportunity occurs (i.e. a new follow-up time point where the intervention could be applied). The main advantages are as follows: (a) it allows the estimation of an average effect across time points, thus increasing generalisability; (b) it can be more efficient than a parallel arm trial due to increased sample size; and (c) it allows subgroup analyses to estimate effectiveness at different time points. We present a case study where the re-randomisation design is used in a SWAT.

**Results:**

In our case study, the host trial is a dental trial with two available follow-up points. The Sticker SWAT tests whether adding the trial logo’s sticker to the questionnaire’s envelope will result in a higher response rate compared with not adding the sticker. The primary outcome is the response rate to postal questionnaires. The re-randomisation design could double the available sample size compared to a parallel arm trial, resulting in the ability to detect an effect size around 28% smaller.

**Conclusion:**

The re-randomisation design can increase the efficiency and generalisability of SWATs for trials with multiple follow-up time points.

## Background

Randomised trials are the gold standard for evaluating the effect of interventions. Poor retention in trials can lead to missing data which has serious consequences for the validity of results. Missing data can be dealt with statistically using methods such as multiple imputation, but such methods are only unbiased under strong, untestable assumptions [1]. As such, missing data should be minimised as much as possible to avoid the potential for bias [2], which can drastically affect trial results [3]. However, missing data remains an issue in trials: up to 50% of all trials lose more than 11% of their participants [4]. For this reason, a substantial amount of work is done using studies within trials (SWATs) to learn the most effective ways to retain participants in a trial [5].

A SWAT is a self-contained research study that has been embedded within a host trial with the aim of evaluating or exploring alternative ways of delivering or organising a particular trial process [6]. Our focus is on SWATs to improve retention, i.e. research studies that evaluate or explore alternative ways designed to maximise data collection from trial participants once they have been recruited and randomised [5]. Often, retention SWATs are evaluated at a single time point only, even if they could be applied to any follow-up time point. In practice, trialist’s interest is likely to be in the effect at any time point when trial data is collected (e.g. if evaluating a text message reminder that would be used for each appointment, we want to know how effective it is when used for each appointment, not just at the first appointment). Parallel arm trials, which are the most common design for retention SWATs [5], assume the intervention effect is the same independent of the time point it is assessed at. However, this assumption might not be realistic, especially considering retention SWATs often test behavioural interventions and their effectiveness can be affected by repeated exposure [7]. As such, alternative trial designs, which allow evaluation of interventions across multiple time-points and exploration of the effect of the intervention at different time points, should be considered.

A re-randomisation design allows re-enrolment and re-randomisation of participants whenever a new retention opportunity occurs [8], where there is potential for the SWAT intervention to be reapplied because a new questionnaire or clinical appointment to collect data is taking place. By allowing participants to be re-enrolled at each new data collection point, re-randomisation designs provide larger sample sizes than parallel group trials and estimate the effect of the intervention each time it is used, rather than only the first time. In this paper, we introduce the re-randomisation design for retention SWATs, present a real-world application in a host trial, and discuss the benefits and limitations of implementing it.

## Methods

### Motivation for re-randomisation trials

Re-randomisation designs have previously been used to evaluate interventions for clinical conditions for which some participants may require treatment on more than one occasion. Examples include sickle cell pain crises [9] (with participants being re-randomised for each new pain crisis), severe asthma exacerbations [10] (participants are re-randomised for each new exacerbation), influenza vaccines [11] (participants are re-randomised each new influenza seasons), in vitro fertilisation [12] (participants are re-randomised for each new cycle), and pre-term birth [13] (participants are re-randomised for each new pregnancy).

Similarly, re-randomisation could be used for SWATs which are evaluating interventions that could be used more than once. For instance, some retention interventions, such as a text message reminder may be used for each new questionnaire issued. When planning a SWAT it is essential to consider a precise description of the treatment effect to be estimated (i.e. what question is the SWAT aiming to address precisely?). This is called an estimand [14].

The main feature of the re-randomisation design is that it allows us to estimate the average effect of the intervention across all retention opportunities for which it would be used in practice, thus providing more generalisable results. For instance, consider a text message reminder to reply to a questionnaire; if found effective, future trials would likely use this intervention as a reminder for each questionnaire issued during the trial, however many questionnaires that might be. Thus, contrary to a parallel group design, which provides the effect of the intervention if used for a single questionnaire, the re-randomisation design allows us to understand how well the intervention works as used in practice, across multiple time points.

Another feature of the re-randomisation design is that it facilitates a larger sample size, as participants can be enrolled for multiple retention opportunities [8, 15, 16]. This can lead to increased efficiency compared to parallel group trials, which results in either the ability to answer the research question faster or the ability to detect smaller differences between the intervention and control arm.

### Implementation

We summarise key considerations to implement re-randomisation designs for SWATs in Table 1. The design requirements for re-randomisation trials are that (a) participants are only re-enrolled once the follow-up period from their previous enrolment is complete; (b) randomisations for the same participant are independent.

**Table 1:** Key considerations to implement a re-randomisation design for a SWAT

**Consideration**	**Explanation**
***Motivation***	In SWATs evaluating interventions to increase retention, there may be multiple time-points where the same intervention could be used. For instance, in a trial with postal questionnaire follow-ups at 3, 6, and 12 months, a text message reminder could be used at all three time points. We will term each timepoint a “retention opportunity”, i.e. an episode or occurrence where trial participants can be retained. Re-randomisation trials enable participants to be re-enrolled and re-randomised for each new retention opportunity (e.g. participants could be re-randomised between a text message reminder vs. usual care for each follow-up point at 3, 6, and 12 months) [8]. By including participants across all retention opportunities, re-randomisation trials provide the average treatment effect across opportunities. This allows trialists to estimate the pragmatic effect of introducing the intervention across all opportunities in practice [17].
***Estimand(s), i.e. what question is the SWAT aiming to answer?***	Re-randomisation trials can be used to estimate the average treatment effect across retention opportunities. They can also be used to estimate the effect at each retention opportunity (e.g. the effect of a text message reminder at 3, 6, and 12 months) [17]. This can be useful to evaluate whether the intervention’s effectiveness changes when it’s been used previously (e.g. due to repeat exposures), which can help provide a more complete understanding of how interventions should be used in practice. Both types of estimand (the average effect across retention opportunities, and the effect at each individual retention opportunity) may be of interest, and so both can be reported (though typically one would be specified as the primary estimand).
***Design requirements***	There are two design requirements for implementing re-randomisation trials [8, 18]: • Participants are only re-enrolled once the follow-up period from their previous enrolment is complete; and • Randomisations for the same participant are independent (i.e. the allocation in a participant’s previous enrolment does not influence their allocation in their subsequent enrolment) Both requirements are to enable unbiased estimation of treatment effects.
***Statistical power***	When the treatment effect is the same across each retention opportunity (e.g. if the intervention improves retention by the same amount at 3, 6, and 12 months), then a re-randomisation trial will have the same statistical power as a parallel group trial with the same number of participants (e.g. a re-randomisation trial with 300 retention opportunities from 100 unique participants would have the same power as a parallel group trial with 300 individual participants who each experience one retention opportunity [8]). However, in SWATs the number of retention opportunities is fixed by the host trial. A parallel group design for the SWAT will only recruit one retention opportunity per participant; however, a re-randomisation design will enrol *all* retention opportunities. Thus, for SWATs when the treatment effect is the same across all retention opportunities, re-randomisation trials will have *more *power than parallel group designs, due to the increased number of retention opportunities enrolled. When the treatment effect is different across retention opportunities (e.g. the intervention is less effective at 6 months compared to at 3 months), then a re-randomisation trial may lose some power; however, it will still typically have higher power than a parallel group design, unless the intervention effect is drastically reduced in subsequent retention opportunities.
***Statistical analysis***	The average treatment effect across retention opportunities can be estimated using “independence estimators”. Independence estimators use an independence working correlation structure, which makes a working assumption that participant outcomes across different retention opportunities are independent. Though this assumption is likely to be false in practice, it is used to ensure unbiased estimation of the treatment effect [17, 18]. Further, independence estimators can be used in conjunction with cluster-robust standard errors, which ensure standard errors are valid even when outcomes across retention opportunities are correlated [18].
***Other considerations related to independence***	Re-randomisation trials require independence for certain aspects of the design, but not others. Here, we describe each of these aspects and clarify whether it is a core requirement for using the re-randomisation design. **1. Randomisations for the same participant are independent** This is a core requirement of re-randomisation trials [8]. It is necessary to ensure that the resulting statistical analyses are unbiased. Importantly, this is not an assumption, but a characteristic of the randomisation procedure, which is set out by investigators, so this requirement can be ensured to hold in practice. **2. Participant outcomes across different retention opportunities are independent** This is not a core requirement of re-randomisation trials. The statistical estimators recommended for re-randomisation trials use a “working” assumption that outcomes are independent, however these methods are unbiased even when this working assumption is incorrect [18]. Further, the use of cluster-robust standard errors can correct for correlation between outcomes in the calculation of the standard errors. **3. The treatment effect is the same across retention opportunities** This is not a core requirement of re-randomisation trials. Unbiased estimates can be obtained regardless of whether the treatment effect is the same at each retention opportunity [17]. However, if the treatment effect does vary, this may increase or decrease the statistical power, depending on whether the effect is larger or smaller in subsequent retention opportunities. This can be accounted for in the sample size or power calculations by modifying the average treatment effect based on how the effect is expected to change across opportunities.

Under requirement (a), the follow-up period for assessment of a SWAT needs to be shorter than the host trial’s follow-up periods. For example, a follow-up questionnaire sent every three months as part of the host trial with a text message reminder SWAT which accompanies the questionnaire needs a follow-up of less than three months (so that the follow-up is complete by the time the next questionnaire is issued). This requirement ensures there are no concurrent enrolments, i.e. that participants are not re-enrolled before data collection for their previous enrolment is complete.

Under requirement (b), randomisations for the same participant must be independent, that is, the participant’s allocation for their first retention opportunity should not influence the intervention arm to which they are allocated for their second retention opportunity (e.g. no forced crossover). This can easily be implemented by not including ‘participant’ as a stratification/minimisation factor in the randomisation procedure. The rationale behind this requirement is that forced crossover between opportunities can induce bias in certain circumstances [8].

Finally, it is important to note that the number of times each participant is enrolled in the SWAT is not usually specified in advance, but depends on how many retention opportunities they experience during the main trial. For instance, a participant may withdraw from a trial mid-way through the follow-up period, and no longer receive questionnaires. Under the re-randomisation design, it is acceptable that some participants might be enrolled in the SWAT at each follow-up visit (so they may be enrolled in the trial three times, if there are three follow-up points or retention opportunities), while other participants are only enrolled for one or two follow-up visits.

### Sample size and power calculations

Sample size and power calculations for re-randomisation trials can be conducted using the same methods as for a parallel group trial, except the sample size applies to the number of retention opportunities rather than the number of participants [8]. For instance, if the sample size called for 300 participants, the re-randomisation design would need to enrol 300 retention opportunities (in a SWAT context, if the intervention is a text reminder, this means to enrol 300 text reminders).

Although the same methods from parallel group designs can be used to implement sample size calculations for re-randomisation trials, care should be taken when choosing the target difference. For instance, if we anticipate the intervention effect might be 10% the first time it is used, but 12% the second time, then the specified target difference should be an average of these two figures, weighted according to the number of first vs. second retention opportunities.

### Analysis

Re-randomisation trials can be analysed using independence estimators, which uses a working independence correlation structure [17]. Broadly, this means that re-randomisation trials can be analysed in the same manner as a parallel group trial would be, for instance, using a linear or logistic regression model which treats each retention opportunity as a separate patient.

Using independence estimators, which make the working assumption there is no correlation between opportunities from the same participant, has been shown to provide unbiased estimates of intervention effect and valid standard errors, even when this assumption is not true [19, 20]. Conversely, methods which directly account for such correlation, such as mixed-effects models or generalised estimating equations, can lead to bias in certain settings and should be avoided [19–22].

Independence estimators can be used in conjunction with cluster-robust standard errors, which modify the standard error to allow for clustering [23]; however, valid results can be obtained from model-based standard errors (i.e. see Kahan et al. [8] and Dunning et al. [24]).

A re-randomisation trial can also explore effectiveness at different time points by doing a subgroup analysis by retention opportunity (e.g. 1^st^ vs. 2^nd^). Because of the smaller sample size, this will naturally have less precision than the SWAT main results.

### Real world application

In this section, we describe the Sticker SWAT [25], which uses the re-randomisation design to investigate improving the response rate to postal follow-up questionnaires within a host randomised controlled trial.

### The host trial: REFLECT (A Randomised controlled trial to Evaluate the effectiveness and cost benefit of prescribing high dose FLuoride toothpaste in preventing and treating dEntal Caries in high-risk older adulTs)

The aim of REFLECT is to evaluate the costs and effectiveness of high-dose fluoride toothpaste prescribed in general dental practices to older individuals with a high risk of tooth decay. Participants are randomly allocated to receive prescriptions for 5000 ppm fluoride toothpaste from their dentist plus usual care vs. usual care only. Patient-reported outcomes are collected at baseline and self-reported via annual postal questionnaires issued yearly over a 3-year follow-up period. Excluding baseline there are three follow-up time points of interest (or “retention opportunities”). More information about REFLECT is available in its published protocol [26].

A lower than anticipated response rate to the annual questionnaires was observed in REFLECT. A theory-informed approach has been incorporated in previous trials [27], using behaviour change techniques to improve response rates to postal questionnaires, assuming returning a trial questionnaire is the target behaviour. One possible behaviour change technique is adding a prompt, such as the trial logo added as a sticker to the envelope used to post the trial questionnaire. The sticker would act as a reminder of the trial and prompt the participant to open the envelope and complete the enclosed questionnaire rather than discarding the unopened envelope as presumed junk mail. The Sticker SWAT, registered in the SWAT repository [25], was first used in the IQuaD dental trial, where a trial logo sticker added to the envelope resulted in a small improvement in response rates compared with an envelope with no sticker [27].

The Sticker SWAT aims to answer the research question: “Does a trial logo sticker policy placed on the outside corner of the envelope improve the return of postal questionnaires when compared to a no sticker policy?”.

**Fig. 1:**
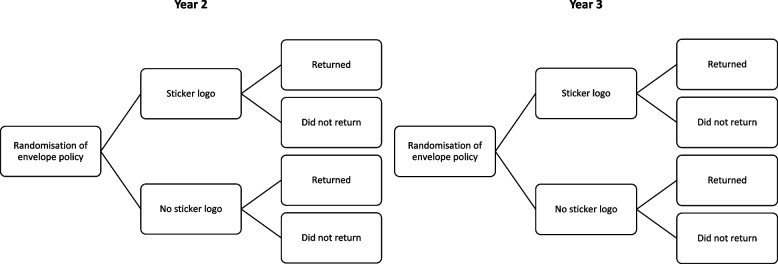
Envelope policy randomisation process in REFLECT’s Sticker SWAT (randomisation happens once in year 2 and once in year 3 of follow-up). All participants taking part in year 2 follow-up will take part in year 3 follow-up unless they explicitly request to withdraw from the trial

The intervention group will receive a trial logo sticker placed on the top corner of the A4 envelope containing the trial questionnaire and cover letter for initial and reminder questionnaires. The control group will receive an A4 envelope containing a questionnaire and cover letter (Comparator) for the initial and reminder questionnaire (Fig. 1).

The Sticker SWAT primary outcome is the response rate to postal questionnaires (defined as the number of questionnaires returned divided by the number of questionnaires sent; this includes both the initial responses and the responses to the reminder). The primary estimand of interest in the Sticker SWAT is the average effect (intervention vs. control) across all retention opportunities (each time a questionnaire is sent out).

The Sticker SWAT fills the re-randomisation design requirements because (a) responses to the questionnaire are accepted and counted for less than a year since its issue (i.e. before participants are eligible to be re-enrolled when the next questionnaire is sent out) and (b) randomisations at each follow-up time point (i.e. at year 2 and year 3) are independent.

### Sample size

**Fig. 2:**
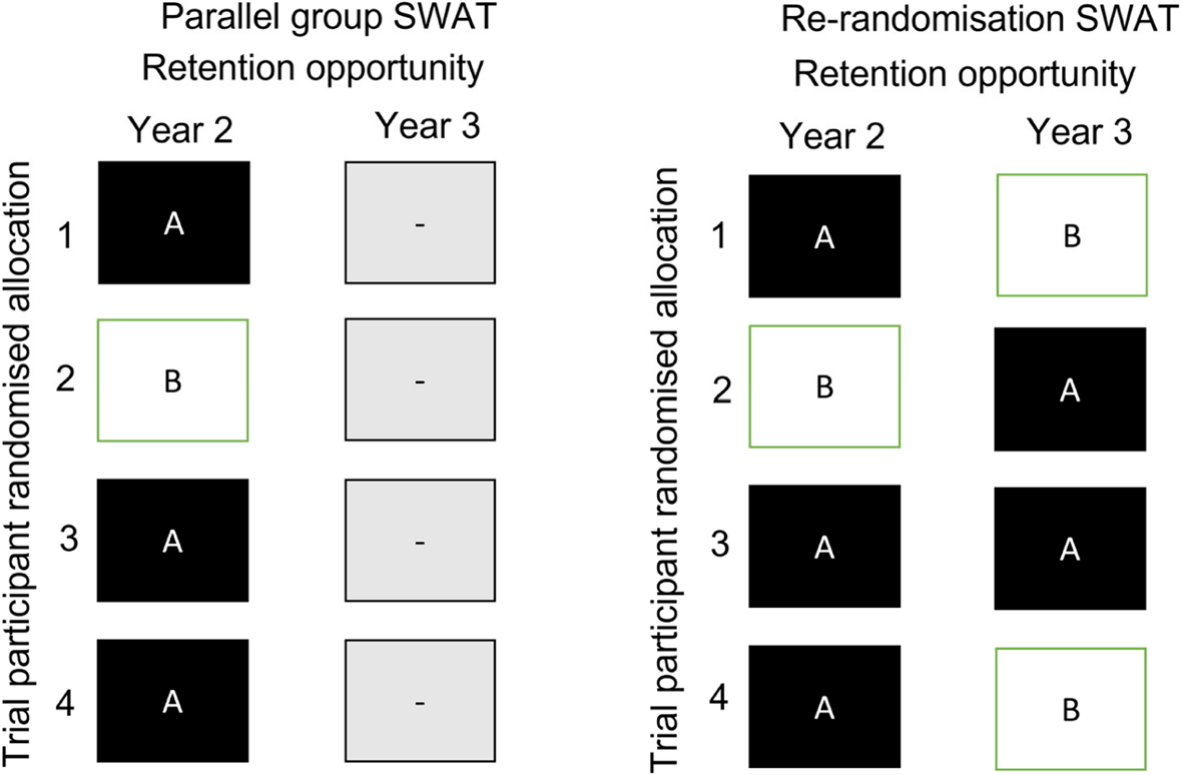
Comparison of a parallel group SWAT and a re-randomisation SWAT for the Sticker Trial in REFLECT. In this context, the “retention opportunities” are the follow-up time points in the host trial

The Sticker SWAT in REFLECT was planned to be implemented in years 2 and 3 of the host trial. Since the host trial has a sample size of 1026 participants, under a re-randomisation design, we would have 2052 questionnaires to send for years 2 and 3 and assuming no drop-out (i.e. participants asking to no longer receive trial questionnaires). In year 1, REFLECT has a 75% response rate. With 2052 total retention opportunities (allocated 1:1, so 1026 in each arm), we have 90% power to detect a 5.9% difference in response rates and 80% power to detect a difference of 5.2% (assuming alpha = 0.05). If we were not using a re-randomisation design, but a parallel arm trial, we would have 90% power to detect an 8.2% difference in response rates, and 80% power to detect a difference of 7.2%. Both sample size calculations are limited by the number of participants (and questionnaires) available in the host trial. Figure 2 highlights the differences between these two design options for the Sticker SWAT in REFLECT.

### Proposed analysis

We will compare the number of letters returned per number of letters sent in each arm and separately by trial using a *Z* test for differences in proportions. We will treat each letter as independent (even letters sent to the same participant at different time-points). A sensitivity analysis using a regression with robust standard errors for participants will be conducted. A subgroup analysis with a treatment-by-time period interaction will explore the effect size of the difference at the different follow-up time points (in our case, year 2 vs year 3).

## Discussion

In this paper, we introduce the re-randomisation design in the context of retention SWATs and present a real-world application that is, to our knowledge, the first example of implementing re-randomisation to test the effectiveness of retention interventions within trials [5]. We argue that re-randomisation designs are a potentially good alternative to parallel arm trials when testing a retention SWAT, when there are multiple retention opportunities. Whether this is the case will be mainly dependent on the SWAT’s estimand (i.e. the exact question being addressed). The re-randomisation design can be a good alternative for three main reasons: (1) the question it answers is potentially more relevant: what is the effect of the retention SWAT over all time points for which it would be used?; (2) it is usually more efficient than a parallel arm trial owing to the increased sample size from randomising retention opportunities instead of individual participants; (3) it enables evaluation of whether the effect of the retention intervention differs across time points.

Using re-randomisation to evaluate a retention SWAT does not necessarily require additional methodological complexity when compared with a parallel arm trial. Often, re-randomisation trials in a clinical context use the same sample size calculation and analysis method as in parallel arm trials, except instead of recruiting and analysing participants, they recruit and analyse treatment episodes [16]. However, there may be additional complexities to re-randomisation, for instance in implementing the randomisation schedule or in communicating the design to stakeholders. Further, the re-randomisation design can be more challenging to interpret (due to the potential to explore ancillary questions) than a parallel arm trial.

Trialists need to consider and be transparent about their assumptions related to the SWAT intervention’s effect over time. This currently appears to be missing from the SWAT literature [5], and we hope the considerations in this paper will help improve that. To generalise the results from a parallel group SWAT trials to general practice (where the intervention might be used over multiple time-points), trialists need to assume the intervention effect is identical each time it is used. Most retention interventions are behavioural [5] and barriers to reply to a questionnaire or attend an appointment may vary during the course of a trial [27]. Behavioural literature shows that interventions might be more likely to work the first time they are implemented [7, 28] rather than subsequent times. This makes the assumption of a constant intervention effect questionable in this context, which has implications for both the choice of design and also to the SWAT intervention implementation if found effective. If the intervention effect is the same each time it is used, the re-randomisation design will give the same answer as a parallel group design, but it is likely to be much more efficient (due to the higher number of retention opportunities enrolled) [8]. This means either getting the answer faster or being able to detect a smaller intervention effect. If the intervention effect varies in different opportunities, then results from re-randomisation trials may be more generalisable than those from parallel group designs, as they apply to all retention opportunities that would occur in practice [18]. Further, re-randomisation trials allow subgroup analyses to be conducted at each retention opportunity to evaluate whether the intervention does vary across time points (though like any subgroup analysis, this will naturally have less precision than the main results).

When using re-randomisation to evaluate a SWAT over multiple retention opportunities, trialists might prefer to include stopping rules in case the intervention appears ineffective. This should be considered on a case-by-case basis and, if deemed appropriate, stopping rules should be established in advance. Just like with clinical trials, early data can mislead and stopping early might not be the best decision [29]; however, in a resource-limited and pressured environment, the risk of continuing to pursue an ineffective strategy might outweigh the need for statistical precision.

The re-randomisation design will not be applicable to scenarios where there is only one time point for data collection or if the time points are close enough in time that enrolment in a SWAT might overlap at each time point. This may be the case in trials that use intensive repeated measures, for example using an area under the curve outcome framework, where each data collection time point might happen within days (or hours) of each other.

## Conclusion

Re-randomisation designs are useful at testing retention interventions and may be more efficient and more relevant than the standard parallel-arm design for SWATs. We recommend that trialists consider re-randomisation when there are multiple retention opportunities.

## Data Availability

Not applicable.
